# Practice patterns in the surgical approach for adolescent varicocelectomy

**DOI:** 10.1186/s40064-015-1573-7

**Published:** 2015-12-14

**Authors:** Miriam Harel, Katherine W. Herbst, Eric Nelson

**Affiliations:** University of Connecticut Health Center, 263 Farmington Avenue, Farmington, CT 06030 USA; Connecticut Children’s Medical Center, 282 Washington Street, Hartford, CT 06106 USA

**Keywords:** Varicocele, Adolescent, Child, Urologic surgical procedures

## Abstract

**Objective:**

To describe practice patterns in the choice of surgical approach for adolescent varicocelectomy using the Pediatric Health Information System (PHIS) database.

**Methods:**

Hospitals enrolled in the PHIS database that reported all outpatient surgeries by CPT code from 2003 to 2012 were included. Patients at least 10 years of age whose records contained both the ICD-9 code for varicocele (456.4) and a CPT code for varicocelectomy [55550 (laparoscopic), 55530 (open inguinal), 55535 (open abdominal)] were identified. Microsurgical approach was identified by the add-on CPT code 69990. Comparisons among surgical approaches were made using one-way ANOVA, and time trend was evaluated with linear regression.

**Results:**

A total cohort of 2528 patients was identified from 38 hospitals. Laparoscopic approach was utilized in 53.6 % of patients. (n = 1354) Microsurgical approach was reported in only 2 % (n = 23) of open varicocelectomies. A subgroup analysis was performed including only those hospitals that reported varicocelectomies in every year of the study period. (n = 587) In this subgroup, 57 % of cases were performed laparoscopically (n = 333), and the trend in laparoscopic cases within this subgroup remained stable over the study period (r^2^ = 0.00, p = 0.97).

**Conclusions:**

Laparoscopic varicocelectomy was the most commonly reported surgical approach in this cohort, and the distribution of surgical approaches appeared to remain stable between 2003 and 2012. While subinguinal microsurgical repair has become the gold standard for management of varicocele in adults with infertility, this technique does not appear to be widely adopted in adolescents, though use of an operating microscope is likely underreported in the PHIS database.

## Background

With an incidence of approximately 15 %, varicocele represents one of the most common surgically correctible urologic anomalies in adolescent males (Diamond 2007). While varicoceles are identified in up to 35 % of men with primary infertility (Mehta and Goldstein 2013), approximately 80 % of adults with varicoceles are asymptomatic and fertile (Diamond et al. 2011). Therefore, one of the major challenges in management of adolescent varicoceles is determining which patients would benefit most from varicocelectomy and at what age (Diamond et al. 2011). While the indications for surgical intervention in these patients are controversial, many experts advocate varicocele repair in patients with a persistent testicular size discrepancy of greater than 20 %, abnormal semen analysis if obtainable, and pain attributable to the varicocele (Diamond et al. 2011).

The ideal surgical approach for adolescent varicocelectomy represents another area of debate. Surgical techniques include an open or laparoscopic abdominal (Palomo) approach, with high ligation of spermatic vascular structures. Alternatively, inguinal (Ivanissevitch) and subinguinal approaches may be utilized, with or without the use of microsurgical techniques (Diamond 2007; Diamond et al. 2011). While the subinguinal microsurgical approach appears to have become the gold standard for varicocele ligation in adult males with infertility due to lower postoperative recurrence and complication rates compared to other techniques (Mehta and Goldstein 2013), this approach has not been widely adopted in the adolescent population.

In this study, we sought to describe practice patterns in the choice of surgical approach for adolescent varicocelectomy using the pediatric health information system (PHIS) database.

## Methods

Data for this study was obtained from the PHIS database, an administrative database that contains inpatient, emergency department, ambulatory surgery, and observation encounter-level data from over 45 not-for-profit, tertiary care pediatric hospitals in the United States. These hospitals are affiliated with the Children’s Hospital Association (Overland Park, KS, USA). Data quality and reliability are assured through a joint effort between the Children’s Hospital Association and participating hospitals. Portions of the data submission and data quality processes for the PHIS database are managed by Truven Health Analytics (Ann Arbor, MI). For the purposes of external benchmarking, participating hospitals provide discharge/encounter data including demographics, diagnoses, and procedures. Nearly all of these hospitals also submit resource utilization data (e.g. pharmaceuticals, imaging, and laboratory) into PHIS. Data are de-identified at the time of data submission, and data are subjected to a number of reliability and validity checks before being included in the database.

Our primary outcome was surgical approach for adolescent varicocelectomy. Hospitals enrolled in the PHIS database that reported outpatient surgeries by Current Procedural Terminology (CPT) code from 2003 to 2012 were included. Since not all procedures are reported by CPT code, the number of procedures reported by the international classification of disease-9 (ICD-9) codes were compared to the number of procedures reported by CPT code for each year. In order to provide quality control of the dataset, hospitals that did not report all procedures by CPT code were excluded for that particular year.

Patients at least 10 years of age whose records contained both the ICD-9 code for varicocele (456.4) and a CPT code for varicocelectomy [55550 (laparoscopic), 55530 (open inguinal), 55535 (open abdominal)] were identified. Microsurgical approach was identified by the add-on CPT code 69990. We attempted to determine the incidence of bilateral intervention by searching the PHIS database for either the billing code appearing twice on a particular patient record or by the CPT modifier code 50. Patients undergoing concurrent hernia or hydrocele repair were excluded, as these additional diagnoses could have impacted the choice of surgical approach for varicocelectomy. In patients who had multiple surgeries for recurrence, only the initial varicocelectomy was included in the analysis.

Comparisons among surgical approaches were made using one-way ANOVA, and time trend was evaluated with linear regression. A subgroup analysis was also performed including only those hospitals that reported varicocelectomy cases for every year in the study period.

## Results

The number of hospitals meeting the inclusion criteria increased from 15 hospitals in 2003 to 33 hospitals in 2012 (Table 1). A total of 38 hospitals were included in this analysis. After excluding 117 patients who underwent concurrent hernia or hydrocele repair, 37 patients younger than 10 years of age, and 78 records from hospitals underreporting ambulatory surgeries by CPT code, a final cohort of 2528 patients was identified. The incidence of bilateral intervention was likely underreported, as none of the records included the billing code twice, and the CPT modifier code 50 was included in only seven records. Therefore, we did not attempt to analyze the effect of bilateral intervention on choice of surgical approach.

**Table 1:** Number of hospitals meeting inclusion criteria per year

Year	Number of hospitals
2003	15
2004	17
2005	17
2006	20
2007	26
2008	24
2009	32
2010	34
2011	33
2012	33

Mean age was 15 years (SD ± 2 years). There was no significant difference in age between the various treatment groups. (p = 0.12) Surgeries were performed by urologists (93 %), general surgeons (6 %), or other/unspecified (1 %). Postoperative infection was reported in 13 records (0.5 %), and other surgical complications were reported in only 2 records (0.1 %).

The majority of patients were Caucasian (72 %), while 6 % were black, 1.5 % were Asian, and the remaining 20 % were other/unspecified. Forty-four percent of patients had private insurance, 20 % had public insurance, and insurance status was not reported in the remaining 36 % of patients.

**Fig. 1:**
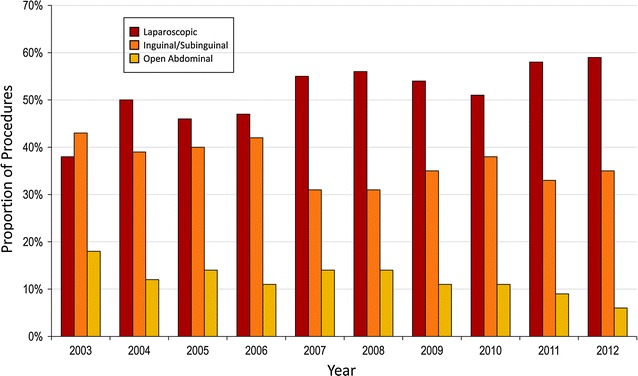
Distribution of varicocelectomies by surgical approach: all hospitals meeting inclusion criteria

Distribution of varicocelectomies by surgical approach is displayed in Fig. 1. Over half of reported varicocelectomies were performed laparoscopically. (n = 1354, 53.6 %) Of the remaining open surgeries, 76.3 % (n = 896) were performed with an inguinal or subinguinal approach, and 23.7 % (n = 278) were approached abdominally. A microsurgical approach was reported in only 2 % (n = 23) of open varicocelectomies (21 inguinal/subinguinal, 2 abdominal). Of the total number of cases, 6 % (n = 147) were performed by general surgeons. In this subgroup, 55 % (n = 81) were performed laparoscopically, 35 % (n = 51) were performed with an inguinal or subinguinal approach, and 10 % (n = 15) were executed with an open abdominal approach. A microsurgical approach was not reported in any of the open varicocelectomies performed by general surgeons. There was no significant difference in the distribution of surgical approaches between urologists and general surgeons.

**Fig. 2:**
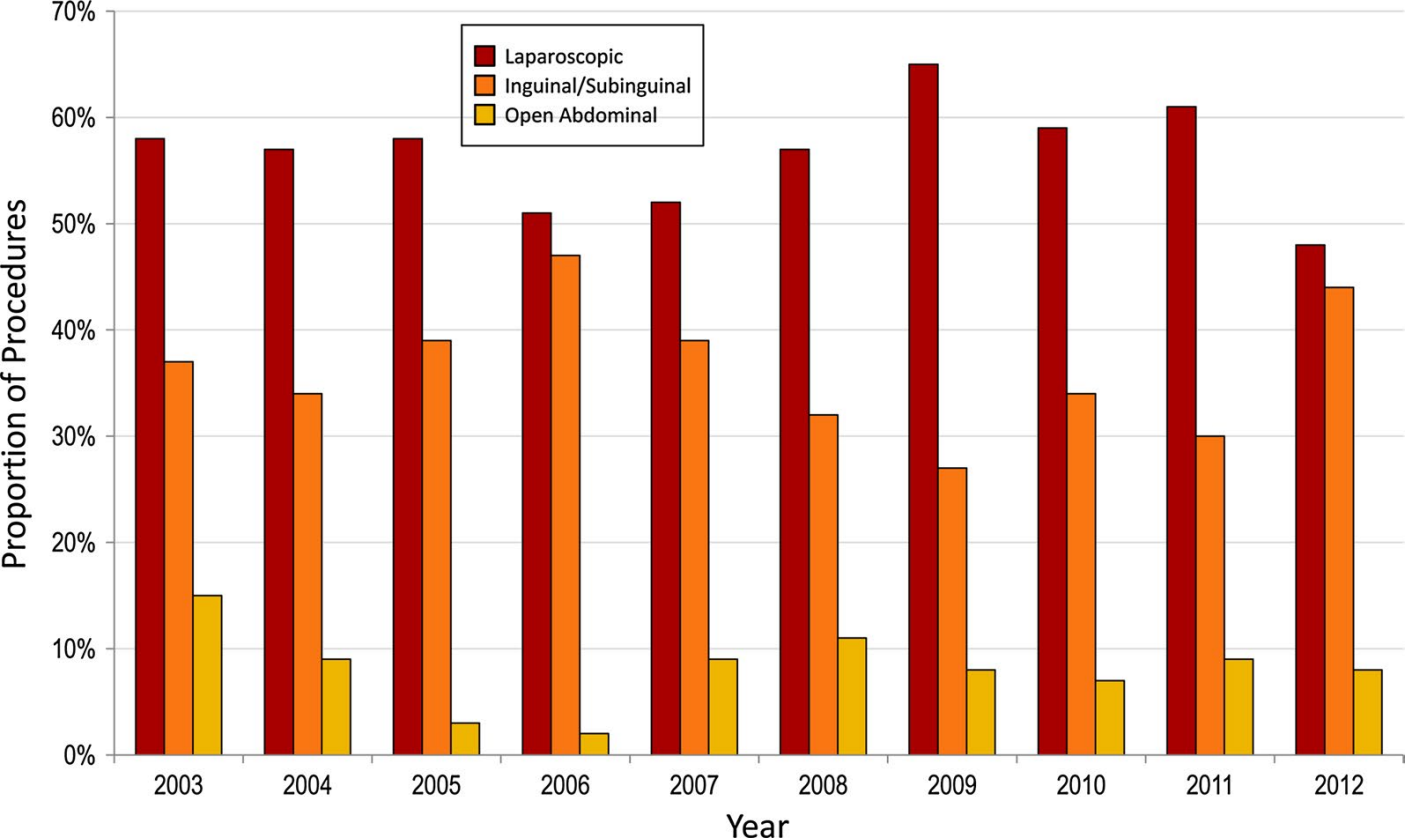
Distribution of varicocelectomies by surgical approach: subgroup analysis

While the proportion of cases performed laparoscopically appeared to increase over the study period (r^2^ = 0.63, p < 0.01), most hospitals did not report cases for every year of the study. Therefore, it was difficult to determine trends using the entire cohort of patients. A subgroup analysis was performed including only those hospitals that reported varicocelectomies in every year of the study period. A total of 587 cases were identified from six reporting hospitals. Distribution of surgical approaches in this subgroup is displayed in Fig. 2. Over half of the cases were performed laparoscopically (n = 333, 57 %), 35 % (n = 206) were performed with an inguinal or subinguinal approach, and the remaining 8 % (n = 48) were performed with an open abdominal approach. In contrast to the findings in the entire cohort, the trend in laparoscopic cases within the subgroup analysis remained stable over the study period (r^2^ = 0.00, p = 0.97).

Each hospital was individually analyzed for distribution of surgical approaches over the study period. Hospitals were considered to favor a certain surgical approach if greater than 50 % of their cases were performed with that particular approach over the entire study period. Of the total 38 hospitals included in the study, 29 % (n = 11) consistently favored a laparoscopic approach throughout the entire study period, 39 % (n = 15) consistently favored an open approach, and 32 % (n = 12) showed variation in surgical techniques over the study period. This suggests that most surgeons may consistently utilize a particular approach, and that the variation in surgical approaches for adolescent varicocelectomy may be explained by surgeon preference.

## Discussion

The ideal surgical technique for adolescent varicocelectomy remains controversial and is usually dependent on surgeon preference. Within the PHIS database, we found that laparoscopic varicocelectomy was the most commonly reported surgical approach in adolescent patients, and that the distribution of surgical approaches appeared to have remained relatively stable from 2003 to 2012. While a subinguinal microsurgical approach has become the gold standard for varicocele ligation in adult males with infertility, a microsurgical approach was reported in only 2 % of children and adolescent patients in the PHIS database.

The most commonly reported risks for any varicocelectomy technique include varicocele recurrence and hydrocele formation. A subinguinal microsurgical approach for varicocele ligation in adult males with infertility has been associated with the lowest postoperative recurrence and complication rates (Mehta and Goldstein 2013). Success rates of 99–100 % have been reported with this technique in adults, with minimal hydrocele rates of 0–0.44 % (Mirilas and Mentessidou 2012; Goldstein et al. 1992). Proponents of this approach report that the magnification of the microscope improves the identification and preservation of the testicular artery and lymphatic vessels and allows for better visualization of all possible routes of venous return (Mehta and Goldstein 2013; Schiff et al. 2005; Lemack et al. 1998).

Several concerns have been raised regarding the application of subinguinal microsurgical varicocelectomy to pediatric patients. Inexperience or lack of familiarity with the microscopic technique is perhaps the most significant obstacle to more widespread adoption of this approach among pediatric urologists (Diamond 2009; Park et al. 2011). Furthermore, complex entanglement of smaller vasculature in pediatric patients is another potential challenge. While testicular atrophy has not been reported after spermatic vein ligation above the internal ring (Diamond et al. 2011), this rare complication has been associated with inguinal and subinguinal approaches (Chan et al. 2005).

Despite these concerns, several groups have performed subinguinal/inguinal microsurgical varicocelectomy in children and adolescents with success rates comparable to those seen in adults (Table 2). No cases of testicular atrophy occurred in these series (Schiff et al. 2005; Lemack et al. 1998; Silveri et al. 2003; Cayan et al. 2005; Yaman et al. 2006). Park et al. (2011) compared the results of microsurgical subinguinal varicocelectomy performed in 31 adults versus 62 adolescents with a mean age of 13 years. In both groups, no cases of recurrence, hydrocele formation, or testicular atrophy were identified. The authors reported a longer operative time and a greater number of almost all components of the venous system in adults, suggesting that this technique may not be more technically challenging in the pediatric population.

**Table 2:** Outcomes of subinguinal/inguinal microsurgical varicocelectomy in children and adolescents

Study	Patients, n	Varicoceles, n	Hydrocele, %	Recurrence, %	Other complications
Lemack et al. (1998)	30	42	0.0	0.0	0.0 %
Silveri et al. (2003)	46 (12 with operating microscope; 34 with loupe magnification)	46	2.1	2.1	4.4 % (wound infections requiring antibiotics)
Schiff et al. (2005)	74	97	1	0.0	2 % (orchalgia in 1 %, resolved after 8 months; hypertrophic scarring in 1 %)
Cayan et al. (2005)	49	79	0.0	0.0	0.0 %
Yaman et al. (2006)	92	92	0.0	1.6	1.6 % (persistent scrotal pain)

Several prior studies have assessed practice patterns in the management of adolescent varicocele using surveys. Richter et al. (2001) reported on questionnaire responses by 99 pediatric urologists and 75 urologists with infertility training. Of the pediatric urologists surveyed, the preferred surgical approaches were inguinal (41.4 %), subinguinal (29.3 %), retroperitoneal Palomo (19.9 %), and laparoscopic (14.4 %). Use of the operating microscope was reported by 30 % of pediatric urologists surveyed. In a more recent survey of 131 pediatric urologists, Pastuszak et al. (2014) reported that the preferred surgical approaches were laparoscopic (38 %), subinguinal microsurgical (28 %), inguinal (14 %), and open Palomo (13 %). Of those urologists who used an open surgical approach, 60 % used loupe magnification, and 40 % used an operating microscope. The authors note that when comparing their survey results to those by Richter et al. (2001), the evaluation and management of pediatric varicocele appears to have remained stable over the past decade, with a shift toward increasing use of a laparoscopic technique. This shift likely reflects an increasing comfort level with the laparoscopic technique among more recent trainees rather than demonstration of superiority of the approach.

There are several possible explanations for the discrepancy in the reported use of a microsurgical approach between these survey studies and our PHIS study. The survey response rate in both aforementioned studies was approximately 50 %, which may limit the generalizability of these results. Additionally, use of the operating microscope is likely underestimated in our study due to underutilization of the 69990 CPT code. However, even if all the inguinal/subinguinal varicocelectomies in our study were performed microsurgically, this approach would comprise approximately 30 % of all cases, which still raises the question of whether a microsurgical approach should be more widely adopted in adolescent patients.

This study has several noteworthy limitations. Incidence of bilateral intervention was not obtainable due to underutilization of the modifier code by participating hospitals. Inconsistent reporting of hospitals for every year in the study period limited our ability to determine trends in the choice of surgical approach for adolescent varicocelectomy. We attempted to determine trends by performing a subgroup analysis including only those hospitals that reported varicocelectomies in every year of the study period; however, the subgroup analysis included less than 25 % of the entire cohort. Furthermore, practices utilized by the tertiary care pediatric hospitals included in the PHIS database may not be representative of practice patterns in the remaining hospitals across the nation.

## Conclusions

Laparoscopic varicocelectomy was the most commonly reported surgical approach in this adolescent cohort. Although it was difficult to determine trends due to inconsistent reporting by CPT code, a subgroup analysis of hospitals reporting by CPT code for all study years suggested that the distribution of surgical approaches remained relatively stable between 2003 and 2012. While subinguinal microsurgical repair has become the gold standard for management of varicocele in adult males with infertility, this technique does not appear to be widely adopted in adolescents, though use of an operating microscope is likely underreported in the PHIS database.

## Abbreviations

PHIS: pediatric health information system
CPT: current procedural terminology
ICD-9: international classification of disease-9
